# Supplementary material for: Identification and Validation of Key Genes Related to Preferred Flavour Profiles in Australian Commercial Papaya (Carica papaya L.)
Source: Int J Mol Sci. 2024 Mar 6;25(5):3046. doi: 10.3390/ijms25053046 (PMC10932401; doi:10.3390/ijms25053046)
Supplement: Supplementary file 1 [file ijms-25-03046-s001.zip › ijms-2850995-supplementary.pdf]

Supplementary material

Table S1. RNA samples quality report.

Sample Name	Peak Count	Concentration (ng/µl)	RNA Area	rRNA Area Ratio [28S/18S]	rRNA Height Ratio [28S/18S]	rRNA Fast Area Ratio	RNA Quality Score	5S Area	5S % Total	18S Area	18S % Total	28S Area	28S % Total
RB1 ripe_1	7	69.56	107.81	1.31	1.04	0.22	6.5	18.69	17.30%	15.22	14.10%	19.97	18.50%
RB1 ripe_2	9	53.23	82.71	1.5	1.01	0.23	6.3	16.19	19.60%	10.53	12.70%	15.8	19.10%
RB1 ripe_3	7	37.22	58.02	1.63	1.02	0.21	6.5	12.8	22.10%	7.51	12.90%	12.27	21.10%
RB1 unripe_1	7	23.62	37.81	1.74	1.13	0.21	6.3	8.06	21.30%	3.82	10.10%	6.66	17.60%
RB1 unripe_2	6	23.1	36.3	1.67	1.03	0.2	6.3	7.44	20.50%	3.84	10.60%	6.4	17.60%
RB1 unripe_3	8	33	53.32	1.6	1.06	0.22	6.5	10.17	19.10%	6.81	12.80%	10.92	20.50%
1B ripe_1	8	49.63	75.08	1.3	0.99	0.22	6.1	16.59	22.10%	9.78	13.00%	12.67	16.90%
1B ripe_2	8	53.99	79.91	1.35	1.12	0.21	6.9	12.44	15.60%	12.88	16.10%	17.39	21.80%
1B ripe_3	9	53.41	78.62	1.29	1.14	0.23	6.2	16.09	20.50%	9.76	12.40%	12.54	16.00%
1B unripe_1	16	88.02	138.98	1.65	1.2	0.13	8.2	9.24	6.70%	23.73	17.10%	39.17	28.20%
1B unripe_2	11	102.44	159.51	1.63	1.16	0.17	8.2	11.25	7.10%	31.09	19.50%	50.66	31.80%
1B unripe_3	20	90.81	139.4	1.73	1.16	0.16	7.6	11.43	8.20%	18.69	13.40%	32.27	23.10%

Table S2. Gene Ontology (GO) enrichment analysis based on DE transcripts between ‘RB1’ and ‘1B’ at ripe stage. (MF = molecular function, CC = cell cycle, BP = biological processes).

ID	p-Value	q-Value	Count	Ontology	Term
GO:0016705	0.000761	0.000693	53	MF	oxidoreductase activity, acting on paired donors, with incorporation or reduction of molecular oxygen
GO:0009317	0.007471	0.006808	10	MF	acetyl-CoA carboxylase complex
GO:0003955	0.004927	0.00449	8	MF	NAD(P)H dehydrogenase (quinone) activity
GO:0047325	0.013537	0.012336	8	MF	inositol tetrakisphosphate 1-kinase activity
GO:0016998	0.000719	0.000655	7	MF	cell wall macromolecule catabolic process
GO:0009579	0.035515	0.032364	7	MF	thylakoid
GO:0008194	0.004223	0.003848	16	CC	UDP-glycosyltransferase activity
GO:0003989	0.004151	0.003782	12	CC	acetyl-CoA carboxylase activity
GO:0009733	0.004927	0.00449	12	CC	response to auxin
GO:0007017	0.031912	0.029081	11	CC	microtubule-based process
GO:0046923	0.00085	0.000775	10	CC	ER retention sequence binding
GO:0120009	0.008117	0.007397	8	CC	intermembrane lipid transfer
GO:0050832	0.010249	0.009339	8	CC	defense response to fungus
GO:0015267	0.030249	0.027565	8	CC	channel activity
GO:0004568	0.000719	0.000655	7	CC	chitinase activity
GO:0009916	0.007471	0.006808	7	CC	alternative oxidase activity
GO:0004362	0.001204	0.001097	6	CC	glutathione-disulfide reductase activity
GO:0004675	0.003794	0.003457	6	CC	transmembrane receptor protein serine/ threonine kinase activity
GO:0005102	0.003794	0.003457	6	CC	signaling receptor binding
GO:0010011	0.012643	0.011521	5	CC	auxin binding
GO:0006559	0.041925	0.038205	5	CC	L-phenylalanine catabolic process
GO:0030001	0.00659	0.006005	29	BP	metal ion transport
GO:0006633	0.005694	0.005189	21	BP	fatty acid biosynthetic process
GO:0046873	0.008117	0.007397	20	BP	metal ion transmembrane transporter activity
GO:0005484	0.01888	0.017205	13	BP	SNAP receptor activity
GO:0006621	0.00085	0.000775	10	BP	protein retention in ER lumen
GO:0010468	0.031912	0.029081	10	BP	regulation of gene expression

GO:0005200	0.021821	0.019885	9	BP	structural constituent of cytoskeleton
GO:0120013	0.008117	0.007397	8	BP	lipid transfer activity
GO:0032957	0.013537	0.012336	8	BP	inositol trisphosphate metabolic process
GO:0052725	0.013537	0.012336	8	BP	inositol-1,3,4-trisphosphate 6-kinase activity
GO:0052726	0.013537	0.012336	8	BP	inositol-1,3,4-trisphosphate 5-kinase activity
GO:0005452	0.031912	0.029081	8	BP	inorganic anion exchanger activity
GO:0006820	0.031912	0.029081	8	BP	anion transport
GO:0006032	0.000719	0.000655	7	BP	chitin catabolic process
GO:0008061	0.00085	0.000775	7	BP	chitin binding
GO:0042744	0.031912	0.029081	7	BP	hydrogen peroxide catabolic process
GO:0032509	0.011469	0.010451	6	BP	endosome transport via multivesicular body sorting pathway

Table S3. The differentially expressed transcripts between 'RB1' and '1B' at ripe stage which are related to sugar and volatile metabolism pathways.

TrinityID	KO Number	Function	Accession	Description
TRINITY_DN1530_c0_g1_i45	K00232	Fatty acid biosynthesis	XM_022042730.1	PREDICTED: Carica papaya transcription factor VOZ2-like (LOC110815073), transcript variant X2, mRNA
TRINITY_DN1530_c0_g1_i96	K00233	Fatty acid biosynthesis	XM_022042730.1	PREDICTED: Carica papaya transcription factor VOZ2-like (LOC110815073), transcript variant X2, mRNA
TRINITY_DN578_c0_g1_i67	K01962	Fatty acid biosynthesis	XM_022041639.1	PREDICTED: Carica papaya acetyl-coenzyme A carboxylase carboxyl transferase subunit alpha, chloroplastic (LOC110814239), mRNA
TRINITY_DN10296_c0_g1_i1	K02160	Fatty acid biosynthesis	XM_022035231.1	PREDICTED: Carica papaya biotin carboxyl carrier protein of acetyl-CoA carboxylase 1, chloroplastic (LOC110809409), mRNA
TRINITY_DN1177_c0_g1_i49	K00963	Starch and sucrose metabolism	XM_022032344.1	PREDICTED: Carica papaya UTP--glucose-1-phosphate uridylyltransferase (LOC110807265), mRNA
TRINITY_DN1177_c0_g1_i55	K00963	Starch and sucrose metabolism	XM_022032344.1	PREDICTED: Carica papaya UTP--glucose-1-phosphate uridylyltransferase (LOC110807265), mRNA
TRINITY_DN12097_c0_g1_i12	K01597	Terpenoid backbone biosynthesis	XM_022032147.1	PREDICTED: Carica papaya diphosphomevalonate decarboxylase MVD2, peroxisomal (LOC110807119), mRNA
TRINITY_DN1228_c0_g1_i9	K00927	Glycolysis / Gluconeogenesis	XM_022033375.1	PREDICTED: Carica papaya leucine--tRNA ligase, chloroplastic/mitochondrial (LOC110808039), mRNA

TRINITY_DN1264_c0_g1_i17	K03526	Terpenoid backbone biosynthesis	XM_022031320.1	PREDICTED: Carica papaya 4-hydroxy-3-methylbut-2-en-1-yl diphosphate synthase (ferredoxin), chloroplastic (LOC110806447), transcript variant X1, mRNA
TRINITY_DN1273_c2_g1_i3	K01193	Starch and sucrose metabolism	XM_022036773.1	PREDICTED: Carica papaya beta-fructofuranosidase, insoluble isoenzyme 1 (LOC110810577), mRNA
TRINITY_DN1273_c2_g1_i4	K01193	Starch and sucrose metabolism	XM_022036773.1	PREDICTED: Carica papaya beta-fructofuranosidase, insoluble isoenzyme 1 (LOC110810577), mRNA
TRINITY_DN1285_c2_g1_i5	K15633	Glycolysis / Gluconeogenesis	XM_022051093.1	PREDICTED: Carica papaya 2,3-bisphosphoglycerate-independent phosphoglycerate mutase (LOC110821299), mRNA
TRINITY_DN1285_c2_g1_i7	K15633	Glycolysis / Gluconeogenesis	XM_022051093.1	PREDICTED: Carica papaya 2,3-bisphosphoglycerate-independent phosphoglycerate mutase (LOC110821299), mRNA
TRINITY_DN1288_c0_g1_i14	K00873	Glycolysis / Gluconeogenesis	XM_022053864.1	PREDICTED: Carica papaya plastidial pyruvate kinase 2 (LOC110823474), transcript variant X2, mRNA
TRINITY_DN1293_c0_g1_i9	K00626	Terpenoid backbone biosynthesis	XM_022031434.1	PREDICTED: Carica papaya acetyl-CoA acetyltransferase, cytosolic 1-like (LOC110806540), mRNA
TRINITY_DN1403_c0_g1_i1	K00128	Glycolysis / Gluconeogenesis	XM_022035172.1	PREDICTED: Carica papaya aldehyde dehydrogenase family 2 member B4, mitochondrial (LOC110809363), mRNA
TRINITY_DN1426_c0_g1_i21	K00688	Starch and sucrose metabolism	XM_022036038.1	PREDICTED: Carica papaya alpha-glucan phosphorylase, H isozyme (LOC110810011), mRNA
TRINITY_DN1426_c0_g1_i22	K00688	Starch and sucrose metabolism	XM_022036038.1	PREDICTED: Carica papaya alpha-glucan phosphorylase, H isozyme (LOC110810011), mRNA
TRINITY_DN1426_c0_g1_i23	K00688	Starch and sucrose metabolism	XM_022036038.1	PREDICTED: Carica papaya alpha-glucan phosphorylase, H isozyme (LOC110810011), mRNA
TRINITY_DN1426_c0_g1_i3	K00688	Starch and sucrose metabolism	XM_022036038.1	PREDICTED: Carica papaya alpha-glucan phosphorylase, H isozyme (LOC110810011), mRNA
TRINITY_DN150_c2_g1_i107	K01188	Starch and sucrose metabolism	XM_022039087.1	PREDICTED: Carica papaya putative beta-glucosidase 41 (LOC110812332), transcript variant X1, mRNA

TRINITY_DN150_c2_g1_i38	K01188	Starch and sucrose metabolism	XM_022039088.1	PREDICTED: Carica papaya putative beta-glucosidase 41 (LOC110812332), transcript variant X2, mRNA
TRINITY_DN150_c2_g1_i42	K01188	Starch and sucrose metabolism	XM_022039088.1	PREDICTED: Carica papaya putative beta-glucosidase 41 (LOC110812332), transcript variant X2, mRNA
TRINITY_DN150_c2_g1_i89	K01188	Starch and sucrose metabolism	XM_022039088.1	PREDICTED: Carica papaya putative beta-glucosidase 41 (LOC110812332), transcript variant X2, mRNA
TRINITY_DN150_c2_g1_i90	K01188	Starch and sucrose metabolism	XM_022039088.1	PREDICTED: Carica papaya putative beta-glucosidase 41 (LOC110812332), transcript variant X2, mRNA
TRINITY_DN15218_c0_g1_i5	K01006	Glycolysis / Gluconeogenesis	XM_022049256.1	PREDICTED: Carica papaya pyruvate, phosphate dikinase, chloroplastic (LOC110819914), mRNA
TRINITY_DN1652_c0_g1_i13	K00700	Starch and sucrose metabolism	XM_022052755.1	PREDICTED: Carica papaya 1,4-alpha-glucan-branching enzyme 2-2, chloroplastic/amyloplastic-like (LOC110822613), mRNA
TRINITY_DN17_c0_g1_i23	K00627	Glycolysis / Gluconeogenesis	XM_022036580.1	PREDICTED: Carica papaya dihydrolipoyllysine-residue acetyltransferase component 2 of pyruvate dehydrogenase complex, mitochondrial-like (LOC110810411), transcript variant X1, mRNA
TRINITY_DN17_c0_g1_i29	K00627	Glycolysis / Gluconeogenesis	XM_022036580.1	PREDICTED: Carica papaya dihydrolipoyllysine-residue acetyltransferase component 2 of pyruvate dehydrogenase complex, mitochondrial-like (LOC110810411), transcript variant X1, mRNA
TRINITY_DN17_c0_g1_i30	K00627	Glycolysis / Gluconeogenesis	XM_022036580.1	PREDICTED: Carica papaya dihydrolipoyllysine-residue acetyltransferase component 2 of pyruvate dehydrogenase complex, mitochondrial-like (LOC110810411), transcript variant X1, mRNA
TRINITY_DN1767_c0_g1_i27	K15889	Terpenoid backbone biosynthesis	XM_022032313.1	PREDICTED: Carica papaya isoprenylcysteine alpha-carbonyl methylesterase ICME (LOC110807238), mRNA
TRINITY_DN1817_c0_g1_i26	K07513	Fatty acid biosynthesis	XM_022041276.1	PREDICTED: Carica papaya 3-ketoacyl-CoA thiolase 2, peroxisomal (LOC110813975), mRNA
TRINITY_DN1831_c0_g1_i1	K01897	Fatty acid biosynthesis	XM_022041039.1	PREDICTED: Carica papaya long chain acyl-CoA synthetase 2 (LOC110813779), mRNA

TRINITY_DN1831_c0_g1_i28	K01897	Fatty acid biosynthesis	XM_022041039.1	PREDICTED: Carica papaya long chain acyl-CoA synthetase 2 (LOC110813779), mRNA
TRINITY_DN1847_c0_g1_i9	K01087	Starch and sucrose metabolism	XM_022046223.1	PREDICTED: Carica papaya probable trehalose-phosphate phosphatase F (LOC110817605), transcript variant X1, mRNA
TRINITY_DN1848_c0_g1_i1	K18857	Glycolysis / Gluconeogenesis	XM_022042917.1	PREDICTED: Carica papaya alcohol dehydrogenase class-P (LOC110815235), mRNA
TRINITY_DN1920_c0_g1_i12	K00128	Glycolysis / Gluconeogenesis	XM_022040167.1	PREDICTED: Carica papaya aldehyde dehydrogenase family 2 member B7, mitochondrial (LOC110813141), transcript variant X2, mRNA
TRINITY_DN1920_c0_g1_i15	K00128	Glycolysis / Gluconeogenesis	XM_022040167.1	PREDICTED: Carica papaya aldehyde dehydrogenase family 2 member B7, mitochondrial (LOC110813141), transcript variant X2, mRNA
TRINITY_DN1920_c0_g1_i16	K00128	Glycolysis / Gluconeogenesis	XM_022040165.1	PREDICTED: Carica papaya aldehyde dehydrogenase family 2 member B7, mitochondrial (LOC110813141), transcript variant X1, mRNA
TRINITY_DN1920_c0_g1_i9	K00128	Glycolysis / Gluconeogenesis	XM_022040167.1	PREDICTED: Carica papaya aldehyde dehydrogenase family 2 member B7, mitochondrial (LOC110813141), transcript variant X2, mRNA
TRINITY_DN1938_c0_g1_i2	K01188	Starch and sucrose metabolism	XM_022038006.1	PREDICTED: Carica papaya beta-glucosidase 42-like (LOC110811486), mRNA
TRINITY_DN2089_c0_g2_i6	K01834	Glycolysis / Gluconeogenesis	XM_022040403.1	PREDICTED: Carica papaya 2,3-bisphosphoglycerate-dependent phosphoglycerate mutase (LOC110813320), mRNA
TRINITY_DN2092_c0_g1_i38	K00695	Starch and sucrose metabolism	XM_022032340.1	PREDICTED: Carica papaya sucrose synthase 5 (LOC110807262), mRNA
TRINITY_DN2093_c0_g1_i2	K03527	Terpenoid backbone biosynthesis	XM_022041064.1	PREDICTED: Carica papaya 4-hydroxy-3-methylbut-2-enyl diphosphate reductase, chloroplastic (LOC110813807), mRNA
TRINITY_DN2110_c2_g1_i1	K00128	Glycolysis / Gluconeogenesis	XM_022040737.1	PREDICTED: Carica papaya aldehyde dehydrogenase family 3 member H1 (LOC110813549), mRNA
TRINITY_DN221_c1_g2_i13	K01792	Glycolysis / Gluconeogenesis	XM_022052324.1	PREDICTED: Carica papaya putative glucose-6-phosphate 1-epimerase (LOC110822259), transcript variant X2, mRNA

TRINITY_DN221_c1_g2_i7	K01792	Glycolysis / Gluconeogenesis	XM_022052322.1	PREDICTED: Carica papaya pentatricopeptide repeat-containing protein At5g66520 (LOC110822258), mRNA
TRINITY_DN2388_c0_g1_i1	K01179	Starch and sucrose metabolism	XM_022048886.1	PREDICTED: Carica papaya endoglucanase 25-like (LOC110819632), transcript variant X1, mRNA
TRINITY_DN2406_c1_g1_i8	K10527	Fatty acid biosynthesis	XM_022049200.1	PREDICTED: Carica papaya glyoxysomal fatty acid beta-oxidation multifunctional protein MFP-a (LOC110819879), mRNA
TRINITY_DN2423_c0_g2_i31	K14066	Terpenoid backbone biosynthesis	XM_022048986.1	PREDICTED: Carica papaya solanesyl diphosphate synthase 3, chloroplastic/mitochondrial-like (LOC110819699), transcript variant X1, mRNA
TRINITY_DN2555_c0_g1_i16	K01087	Starch and sucrose metabolism	XM_022044971.1	PREDICTED: Carica papaya probable trehalose-phosphate phosphatase D (LOC110816680), mRNA
TRINITY_DN260_c0_g1_i5	K00705	Starch and sucrose metabolism	XM_022031758.1	PREDICTED: Carica papaya 4-alpha-glucanotransferase DPE1, chloroplastic/amyloplastic (LOC110806815), mRNA
TRINITY_DN260_c0_g1_i63	K00705	Starch and sucrose metabolism	XM_022031758.1	PREDICTED: Carica papaya 4-alpha-glucanotransferase DPE1, chloroplastic/amyloplastic (LOC110806815), mRNA
TRINITY_DN260_c0_g1_i98	K00705	Starch and sucrose metabolism	XM_022031758.1	PREDICTED: Carica papaya 4-alpha-glucanotransferase DPE1, chloroplastic/amyloplastic (LOC110806815), mRNA
TRINITY_DN2620_c0_g1_i44	K17982	Diterpenoid biosynthesis	XM_022046578.1	PREDICTED: Carica papaya (E,E)-geranyllinalool synthase-like (LOC110817861), mRNA
TRINITY_DN2620_c0_g1_i57	K22049	Monoterpenoid biosynthesis	XM_022049165.1	PREDICTED: Carica papaya S-linalool synthase-like (LOC110819841), partial mRNA
TRINITY_DN2802_c0_g1_i49	K00895	Glycolysis / Gluconeogenesis	XM_022039277.1	PREDICTED: Carica papaya pyrophosphate--fructose 6-phosphate 1-phosphotransferase subunit beta (LOC110812486), mRNA
TRINITY_DN3009_c0_g1_i2	K01188	Starch and sucrose metabolism	XM_022053230.1	PREDICTED: Carica papaya beta-glucosidase 31-like (LOC110822982), mRNA
TRINITY_DN3108_c0_g1_i2	K05349	Starch and sucrose metabolism	XM_022036929.1	PREDICTED: Carica papaya beta-glucosidase BoGH3B-like (LOC110810687), mRNA
TRINITY_DN3108_c0_g1_i3	K05349	Starch and sucrose metabolism	XM_022036929.1	PREDICTED: Carica papaya beta-glucosidase BoGH3B-like (LOC110810687), mRNA

TRINITY_DN3108_c0_g1_i7	K05349	Starch and sucrose metabolism	XM_022036929.1	PREDICTED: Carica papaya beta-glucosidase BoGH3B-like (LOC110810687), mRNA
TRINITY_DN3108_c0_g1_i8	K05349	Starch and sucrose metabolism	XM_022036929.1	PREDICTED: Carica papaya beta-glucosidase BoGH3B-like (LOC110810687), mRNA
TRINITY_DN31090_c0_g1_i1	K00128	Glycolysis / Gluconeogenesis	XM_022052648.1	PREDICTED: Carica papaya aldehyde dehydrogenase family 3 member F1 (LOC110822516), mRNA
TRINITY_DN3366_c0_g1_i1	K00002	Glycolysis / Gluconeogenesis	XM_022045556.1	PREDICTED: Carica papaya NADPH-dependent aldo-keto reductase, chloroplastic-like (LOC110817140), mRNA
TRINITY_DN3457_c0_g1_i1	K01193	Starch and sucrose metabolism	XM_022053955.1	PREDICTED: Carica papaya acid beta-fructofuranosidase 1, vacuolar-like (LOC110823556), mRNA
TRINITY_DN3457_c0_g1_i2	K01193	Starch and sucrose metabolism	XM_022053955.1	PREDICTED: Carica papaya acid beta-fructofuranosidase 1, vacuolar-like (LOC110823556), mRNA
TRINITY_DN3547_c0_g1_i101	K01805	Fructose and mannose metabolism	XM_022037464.1	PREDICTED: Carica papaya xylose isomerase (LOC110811088), transcript variant X5, mRNA
TRINITY_DN3547_c0_g1_i129	K01805	Fructose and mannose metabolism	XM_022037460.1	PREDICTED: Carica papaya xylose isomerase (LOC110811088), transcript variant X1, mRNA
TRINITY_DN3547_c0_g1_i28	K01805	Fructose and mannose metabolism	XM_022037469.1	PREDICTED: Carica papaya xylose isomerase (LOC110811088), transcript variant X9, mRNA
TRINITY_DN4138_c2_g1_i5	K16055	Starch and sucrose metabolism	XM_022048897.1	PREDICTED: Carica papaya alpha,alpha-trehalose-phosphate synthase [UDP-forming] 1-like (LOC110819639), transcript variant X1, mRNA
TRINITY_DN4424_c0_g1_i18	K01179	Starch and sucrose metabolism	XM_022042964.1	PREDICTED: Carica papaya glycolipid transfer protein 3 (LOC110815264), mRNA
TRINITY_DN4424_c0_g1_i5	K01179	Starch and sucrose metabolism	XM_022042964.1	PREDICTED: Carica papaya glycolipid transfer protein 3 (LOC110815264), mRNA
TRINITY_DN451_c0_g1_i129	K01785	Glycolysis / Gluconeogenesis	XM_022053630.1	PREDICTED: Carica papaya aldose 1-epimerase (LOC110823266), mRNA
TRINITY_DN4654_c1_g1_i1	K19355	Fructose and mannose metabolism	XM_022052403.1	PREDICTED: Carica papaya mannan endo-1,4-beta-mannosidase 7 (LOC110822316), mRNA
TRINITY_DN4683_c0_g1_i2	K05906	Terpenoid backbone biosynthesis	XM_022044825.1	PREDICTED: Carica papaya farnesylcysteine lyase (LOC110816574), partial mRNA

TRINITY_DN4930_c0_g1_i18	K00927	Glycolysis / Gluconeogenesis	XM_022041620.1	PREDICTED: Carica papaya phosphoglycerate kinase-like (LOC110814219), transcript variant X6, mRNA
TRINITY_DN5002_c0_g1_i10	K18660	Fatty acid biosynthesis	XM_022056584.1	PREDICTED: Carica papaya malonate--CoA ligase-like (LOC110826011), mRNA
TRINITY_DN5108_c1_g1_i1	K01962	Fatty acid biosynthesis	XM_022048755.1	PREDICTED: Carica papaya acetyl-coenzyme A carboxylase carboxyl transferase subunit alpha, chloroplastic-like (LOC110819518), transcript variant X2, mRNA
TRINITY_DN5108_c1_g1_i2	K01962	Fatty acid biosynthesis	XM_022048754.1	PREDICTED: Carica papaya acetyl-coenzyme A carboxylase carboxyl transferase subunit alpha, chloroplastic-like (LOC110819518), transcript variant X1, mRNA
TRINITY_DN5126_c0_g1_i17	K01176	Starch and sucrose metabolism	XM_022052879.1	PREDICTED: Carica papaya alpha-amylase 3, chloroplastic (LOC110822706), mRNA
TRINITY_DN5126_c0_g1_i33	K01176	Starch and sucrose metabolism	XM_022052879.1	PREDICTED: Carica papaya alpha-amylase 3, chloroplastic (LOC110822706), mRNA
TRINITY_DN5126_c0_g1_i54	K01176	Starch and sucrose metabolism	XM_022052879.1	PREDICTED: Carica papaya alpha-amylase 3, chloroplastic (LOC110822706), mRNA
TRINITY_DN5126_c0_g1_i74	K01176	Starch and sucrose metabolism	XM_022052879.1	PREDICTED: Carica papaya alpha-amylase 3, chloroplastic (LOC110822706), mRNA
TRINITY_DN5126_c0_g1_i77	K01176	Starch and sucrose metabolism	XM_022052879.1	PREDICTED: Carica papaya alpha-amylase 3, chloroplastic (LOC110822706), mRNA
TRINITY_DN5471_c0_g1_i16	K00873	Glycolysis / Gluconeogenesis	XM_022036686.1	PREDICTED: Carica papaya pyruvate kinase isozyme A, chloroplastic-like (LOC110810485), mRNA
TRINITY_DN5472_c0_g1_i11	K00626	Terpenoid backbone biosynthesis	XM_022046537.1	PREDICTED: Carica papaya probable acetyl-CoA acetyltransferase, cytosolic 2 (LOC110817825), transcript variant X2, mRNA
TRINITY_DN5599_c0_g1_i146	K01214	Starch and sucrose metabolism	XM_022044938.1	PREDICTED: Carica papaya isoamylase 3, chloroplastic (LOC110816655), transcript variant X3, mRNA
TRINITY_DN578_c0_g1_i20	K01962	Fatty acid biosynthesis	XM_022041639.1	PREDICTED: Carica papaya acetyl-coenzyme A carboxylase carboxyl transferase subunit alpha, chloroplastic (LOC110814239), mRNA
TRINITY_DN5853_c0_g2_i2	K13679	Starch and sucrose metabolism	XM_022044776.1	PREDICTED: Carica papaya granule-bound starch synthase 1, chloroplastic/amyloplastic (LOC110816544), mRNA

TRINITY_DN6052_c0_g1_i13	K01177	Starch and sucrose metabolism	XM_022048628.1	PREDICTED: Carica papaya beta-amylase 7 (LOC110819443), transcript variant X1, mRNA
TRINITY_DN6052_c0_g1_i15	K01177	Starch and sucrose metabolism	XM_022048628.1	PREDICTED: Carica papaya beta-amylase 7 (LOC110819443), transcript variant X1, mRNA
TRINITY_DN6052_c0_g1_i9	K01177	Starch and sucrose metabolism	XM_022048628.1	PREDICTED: Carica papaya beta-amylase 7 (LOC110819443), transcript variant X1, mRNA
TRINITY_DN6161_c0_g1_i2	K00873	Glycolysis / Gluconeogenesis	XM_022052474.1	PREDICTED: Carica papaya pyruvate kinase isozyme G, chloroplastic (LOC110822371), transcript variant X2, mRNA
TRINITY_DN619_c0_g1_i101	K01662	Terpenoid backbone biosynthesis	XM_022056540.1	PREDICTED: Carica papaya cell division cycle 5-like protein (LOC110825983), mRNA
TRINITY_DN619_c0_g1_i183	K01662	Terpenoid backbone biosynthesis	XM_022037819.1	PREDICTED: Carica papaya probable 1-deoxy-D-xylulose-5-phosphate synthase, chloroplastic (LOC110811343), mRNA
TRINITY_DN6343_c0_g1_i9	K01897	Fatty acid biosynthesis	XM_022039126.1	PREDICTED: Carica papaya long chain acyl-CoA synthetase 4-like (LOC110812363), mRNA
TRINITY_DN638_c1_g1_i14	K03921	Fatty acid biosynthesis	XM_022042916.1	PREDICTED: Carica papaya stearoyl-[acyl-carrier-protein] 9-desaturase 6, chloroplastic-like (LOC110815234), mRNA
TRINITY_DN6534_c0_g3_i28	K00850	Glycolysis / Gluconeogenesis	XM_022052063.1	PREDICTED: Carica papaya ATP-dependent 6-phosphofructokinase 6-like (LOC110822059), mRNA
TRINITY_DN6873_c0_g1_i7	K01662	Terpenoid backbone biosynthesis	XM_022041737.1	PREDICTED: Carica papaya probable 1-deoxy-D-xylulose-5-phosphate synthase 2, chloroplastic (LOC110814317), partial mRNA
TRINITY_DN6957_c0_g1_i6	K01610	Glycolysis / Gluconeogenesis	XM_022034785.1	PREDICTED: Carica papaya phosphoenolpyruvate carboxykinase (ATP)-like (LOC110809076), mRNA
TRINITY_DN6957_c0_g1_i75	K01610	Glycolysis / Gluconeogenesis	XM_022034785.1	PREDICTED: Carica papaya phosphoenolpyruvate carboxykinase (ATP)-like (LOC110809076), mRNA
TRINITY_DN7033_c0_g1_i4	K00975	Starch and sucrose metabolism	XM_022041840.1	PREDICTED: Carica papaya glucose-1-phosphate adenylyltransferase large subunit, chloroplastic/amyloplastic-like (LOC110814392), mRNA

TRINITY_DN7518_c0_g1_i2	K01074	Fatty acid biosynthesis	XM_022037952.1	PREDICTED: Carica papaya palmitoyl-protein thioesterase 1-like (LOC110811440), mRNA
TRINITY_DN7525_c0_g1_i5	K10703	Fatty acid biosynthesis	XM_022041875.1	PREDICTED: Carica papaya very-long-chain (3R)-3-hydroxyacyl-CoA dehydratase PASTICCINO 2B-like (LOC110814409), mRNA
TRINITY_DN7525_c0_g1_i76	K10703	Fatty acid biosynthesis	XM_022041872.1	PREDICTED: Carica papaya guanosine nucleotide diphosphate dissociation inhibitor At5g09550 (LOC110814407), mRNA
TRINITY_DN775_c0_g1_i1	K00695	Starch and sucrose metabolism	XM_022042685.1	PREDICTED: Carica papaya sucrose synthase 2 (LOC110815045), mRNA
TRINITY_DN775_c0_g2_i11	K00695	Starch and sucrose metabolism	XM_022033485.1	PREDICTED: Carica papaya sucrose synthase 3-like (LOC110808116), mRNA
TRINITY_DN775_c0_g2_i2	K00695	Starch and sucrose metabolism	XM_022033485.1	PREDICTED: Carica papaya sucrose synthase 3-like (LOC110808116), mRNA
TRINITY_DN775_c0_g2_i7	K00695	Starch and sucrose metabolism	XM_022033485.1	PREDICTED: Carica papaya sucrose synthase 3-like (LOC110808116), mRNA
TRINITY_DN775_c0_g3_i2	K00695	Starch and sucrose metabolism	XM_022038670.1	PREDICTED: Carica papaya sucrose synthase (LOC110812012), mRNA
TRINITY_DN7757_c0_g1_i8	K00873	Glycolysis / Gluconeogenesis	XM_022052945.1	PREDICTED: Carica papaya pyruvate kinase 1, cytosolic-like (LOC110822754), mRNA
TRINITY_DN807_c0_g1_i8	K01623	Glycolysis / Gluconeogenesis	XM_022035479.1	PREDICTED: Carica papaya fructose-bisphosphate aldolase 6, cytosolic (LOC110809598), mRNA
TRINITY_DN81_c0_g1_i1	K01568	Glycolysis / Gluconeogenesis	XM_022051928.1	PREDICTED: Carica papaya pyruvate decarboxylase 1 (LOC110821947), mRNA
TRINITY_DN893_c2_g1_i31	K00703	Starch and sucrose metabolism	XM_022049000.1	PREDICTED: Carica papaya probable starch synthase 4, chloroplastic/amyloplastic (LOC110819709), transcript variant X1, mRNA
TRINITY_DN9474_c0_g1_i12	K00162	Glycolysis / Gluconeogenesis	XM_022053275.1	PREDICTED: Carica papaya pyruvate dehydrogenase E1 component subunit beta, mitochondrial (LOC110823011), transcript variant X7, mRNA
TRINITY_DN970_c0_g1_i2	K00787	Terpenoid backbone biosynthesis	XM_022046648.1	PREDICTED: Carica papaya farnesyl pyrophosphate synthase 1 (LOC110817926), mRNA

TRINITY_DN9787_c0_g1_i1	K19891	Starch and sucrose metabolism	XM_022054221.1	PREDICTED: Carica papaya glucan endo-1,3-beta-glucosidase 3 (LOC110823757), mRNA
TRINITY_DN4842_c0_g1_i1	K19861	Benzenoid and benzoic acid biosynthesis	XM_022055614.1	PREDICTED: Carica papaya benzyl alcohol O-benzoyltransferase-like (LOC110825152), mRNA

Table S4. Primers used for qRT-PCR in ‘RB1’ and ‘1B’ papaya varieties

**Gene ID**	**Accession**	**Primer Sequence (5’→3’)**	**Primer Tm (°C)**	**Expected Size (bp)**
EF2	JQ678771	F: CTTTGCCTTCGGTCGTGTCTTC<br>R: CACTGTCTCCTGCTTCTTTCCC	82	154
GAPDH	JQ678772	F: CTTTGTTGGTGACAGCAGG<br>R: GGACAGAGGCAATGTACC	80	149
cpBGLU42	XM_022038006.1	F: TCCTTGCATTGCCCAACAACTC<br>R: ACTGCCACATCACCATTGCTTC	62	183
cpBGLU31	XM_022053230.1	F: CCCGGTGGAAAATTAAGCAAAGG<br>R: ACCTTTATACTCCTCCTCGAGAACC	62	150
cpBGH3B	XM_022036929.1	F: TCCTGTCAATACCCTCCCAATCTG<br>R: GCTGGAATGGAAAGAAGATGCAC	61	123
cpPFP	XM_022039277.1	F: TCAAGGGCAGCACATTGTATGG<br>R: AACCACCCTGATTTCTGTATGGG	62	111
cpSUS	XM_022038670.1	F: ATGAAGGCAGCAGCTTGATCAG<br>R: TTCCGACATTTGCCACTTGCC	61	106
cpGES	XM_022046578.1	F: TGTTTGAAGGGTGAAGCAACGC<br>R: TTCAAAGCCATGGAAGCAGCAG	61	179
cpLIS	XM_022049165.1	F: AACCGCGCAGTTATACAAGGAC<br>R: CGCCAAACATAAGATCCGTTGC	61	188
cpBAO	XM_022055614.1	F: AACTGCGTGTCTATGGCGTTG<br>R: AGCAACAGCAAAAGCGAACC	62	151
